# Head and neck squamous cell carcinoma in young patients: a 26-year clinicopathologic retrospective study in a Brazilian specialized center

**DOI:** 10.4317/medoral.23461

**Published:** 2020-03-06

**Authors:** Saygo Tomo, Sebastião Conrado Neto, Francisco Urbano Collado, Maria Lúcia Marçal Mazza Sundefeld, Daniel Galera Bernabé, Éder Ricardo Biasoli, Glauco Issamu Miyahara

**Affiliations:** 1 Oral Oncology Center, Araçatuba Dental School, São Paulo State University - UNESP, Brazil

## Abstract

**Background:**

To describe the clinicopathologic profile of young patients with head and neck squamous cell carcinoma (HNSCC) and compare to middle-aged and elderly adults.

**Material and Methods:**

Patients’ individual records were reviewed for clinicopathologic data. Eighty-nine patients with age 18-45 years old met the inclusion criteria of the study. Two additional groups of middle-aged (n=89) and old (n=89) adults were set to comparative analysis.

**Results:**

Young patients represented 11.9% of all patients diagnosed with HNSCC. Women were more affected by HNSCC in the young and elder groups (*p*= 0.04), and young patients were more prone to be non-smokers (*p*= 0.01) and have lymph node metastasis at the time of diagnosis (*p*=0.04). In the young group, patients diagnosed with the disease in advanced stages were more prone to have a positive familial history of cancer (*p*= 0.04), a positive status of alcohol consumption (*p*= 0.03), and to be heavy drinkers (*p*= 0.01). Survival was not different for the young group in comparison to the other groups.

**Conclusions:**

HNSCC in young patients had a different profile when compared to older patients, especially regarding sex and exposure to the classic risk factors for this disease. The survival of the young group is similar to the older groups and advanced clinical stage is predictor of worse survival.

** Key words:**Mouth neoplasms, young adult, epidemiology.

## Introduction

Head and neck squamous cell carcinoma (HNSCC) is one of the most common human cancers and has a poor prognosis, representing an important public health problem worldwide (1,2). The profile of people affected by HNSCC is classically represented by men older than 50-years old (3). Incidence rates and the clinicopathologic profile of patients affected by HNSCC may vary among different geographic regions (4). These differences might be due to different behavioral profiles regarding the risk factors for the disease (tobacco and alcohol) in different cultures (4).

HNSCC has a low incidence in people younger than 45-years-old (5,6). This is possibly explained by the fact that young patients are not exposed to tobacco and alcohol in the same intensity and duration as older patients (5,6). Based on the etiology and disease behavior, HNSCC in young people is considered a distinct pathologic entity by some researchers (6,7). Thus, other etiologic factors for this disease have been investigated in the young population with HNSCC (3). A higher prevalence of human papillomavirus (HPV) in HNSCC samples from young people compared to older has been reported (8-10). Despite many studies described the association of HNSCC in young people to other risk factors, most patients of this age-group are also exposed to tobacco and alcohol consumption (11-13).

In addition to understand the etiology of HNSCC in young people, the analysis of factors involved in their prognosis is of great relevance. Van Monsjou *et al*. (14) showed that disease-specific survival (DSS) did not differ between young and elderly individuals with HNSCC. However, overall survival (OS) was significantly better for young patients. HPV-positive HNSCC cases had a better prognosis compared to HPV-negative cases (15), which seems to be more common among young patients. On the other hand, further evidence demonstrates a worse prognosis for young patients with HNSCC in comparison to that of elderly patients, suggesting a higher aggressiveness of the disease in the young (16).

There is an evident need for a better understanding of the occurrence of HNSCC in young patients regarding its etiologic background, clinicopathologic characteristics and prognosis in different populations. Thus, in this paper, we describe the clinicopathologic characteristics and prognostic profile of young patients with HNSCC and compare it to those of middle-aged and elderly patients.

## Material and Methods

- Study design

The study population was composed of all patients presenting for diagnosis and treatment of HNSCC from January 1991 through December 2016 at the Oral Oncology Center, Araçatuba Dental School, São Paulo State University - UNESP, Brazil. Patients were included in the study if they had a diagnosis of oral (oral cavity) or oropharyngeal (oropharynx, hypopharynx, and larynx) squamous cell carcinoma (SCC). Exclusion criteria were: have recurrent HNSCC, had undergone previous oncologic treatment, had a histopathologic diagnosis of other diseases, and with tumors in the lips. The study group was composed of 89 patients younger than or 45-years-old diagnosed with HNSCC. A group composed of middle-aged HNSCC patients (46 to 59-years-old) and other composed of elderly patients (>60-years-old) were selected for comparison with the younger group. To avoid skewness, both middle-aged and elderly groups were limited to 89 patients which were set by a systematic sampling system (17).

- Variables and data collection

The relevant variables were collected from the individual medical records of each patient, and the age-group variable was defined as the predictor variable. The following variables were defined as outcome variables: sex, race, location of the primary tumor, status and intensity of tobacco and alcohol consumption, familiar history of cancer, clinical stage according to the American Joint Committee on Cancer (AJCC), histologic grading as recommended by the World Health Organization (WHO), and survival time (in days).

The status of tobacco smoking and alcohol drinking were classified as: current smokers/drinkers and never-smokers/drinkers. The intensity of these habits was graded according to daily consumption in light (<10 cigarettes / 1 alcohol dose), moderate (11-20 cigarettes / 2 – 3 alcohol doses) and severe (>21 cigarettes / >4 alcohol doses) as previously recommended (18). Former smokers/drinkers and current smokers/drinkers were grouped in the same group as patients ever exposed to these risk factors. For statistics purposes, the clinical stage at the time of diagnosis was classified as: early stages (clinical stages I and II) and advanced stages (III and IV).

- Statistical analysis

Data was transferred to electronic Table software (Microsoft Excel 2016®), and statistical analysis was performed using EpiInfo 7.0 (Center for Diseases Control) software. Chi-square and Fisher’s exact tests were used to compare the clinicopathological characteristics among the different HNSCC patients grouped by the age groups. Additionally, the same tests were used to assess the relationship of clinicopathologic characteristics with the disease stage. To analyze the overall survival and disease-specific survival, survival time from the HNSCC patients treated between 1999 and 2011 were included, and Kapplan-Meier method and log-rank test were used. *p-value*s <0.05 were considered statistically significant.

## Results

- Epidemiology

A retrospective analysis of the patients’ files provided a total of 899 patients with HNSCC (excluding lip tumors), among which 107 (11.9%) were younger than 45-years-old. Of those, 89 patients met the inclusion criteria of the study. The middle-aged and elderly groups were adjusted to the number of young patients. Therefore, 89 patients were included in each age group. The annual incidence was calculated based on all files of the Oral Oncology Center, as demonstrated in Table 1. The higher incidence rates of HNSCC among young patients were observed in the years 1996 and 1997 (22.2%), and w null incidence was observed in the year 2008. Neither constant increase nor decrease in the incidence of HNSCC in young people was observed along the last 26 years.

- Comparison of age groups

In the young group, 78 (87.6%) patients were men and 11 (12.4%) were women, while 85 (95.5%) from the middle-aged were men and 4 (4.5%) were women, and in the old-aged group 75 (84.3%) were men, and 14 (15.7%) were women, demonstrating that women were statistically more frequently affected by HNSCC in the young and elder groups than in the middle-aged group (*p*=0.04) (Table 2). Young patients were more likely to be neve-smokers (*p*= 0.01), to have tumors in the oral cavity (*p*= 0.04), and to have lymph node metastasis at the time of diagnosis (*p*= 0.04) (Table 2).

**Figure 1:**
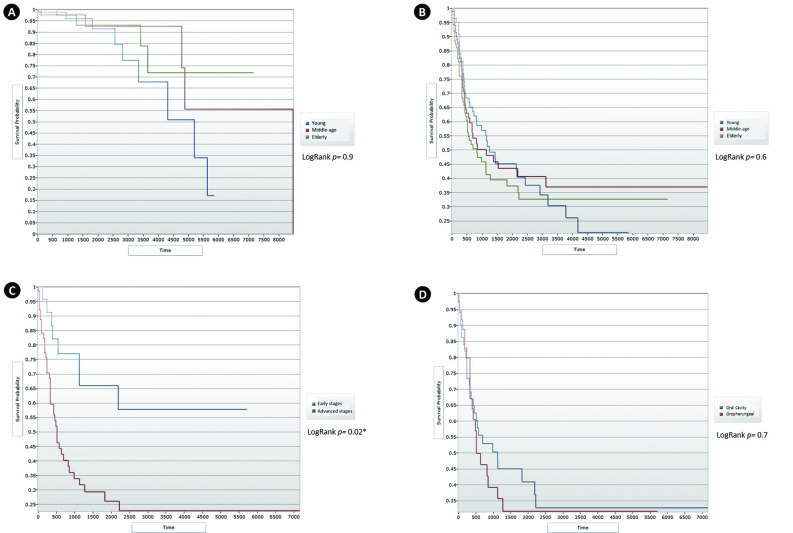
(A) Overall survival (OS) for the three age groups (*p*= 0.9). (B) Disease-specific survival (DSS) for the three age groups (*p*= 0.6). (C) Disease-specific survival (DSS) of young patients according to clinical stage (*p*= 0.02). (D) Disease specific survival (DSS) for oral and oropharyngeal squamous cell carcinoma in the young patients (*p*= 0.7).

Race, familial history of cancer, tobacco smoking intensity, alcohol consumption status and intensity, sublocations of the oral cavity, tumor size, presence of distant metastasis at the time of diagnosis, clinical stage and histologic grading of tumors did not differ among the groups (Table 2). Neither overall survival nor disease-specific survival significantly differed among the age-groups (Fig. 1).

**Table 1:**
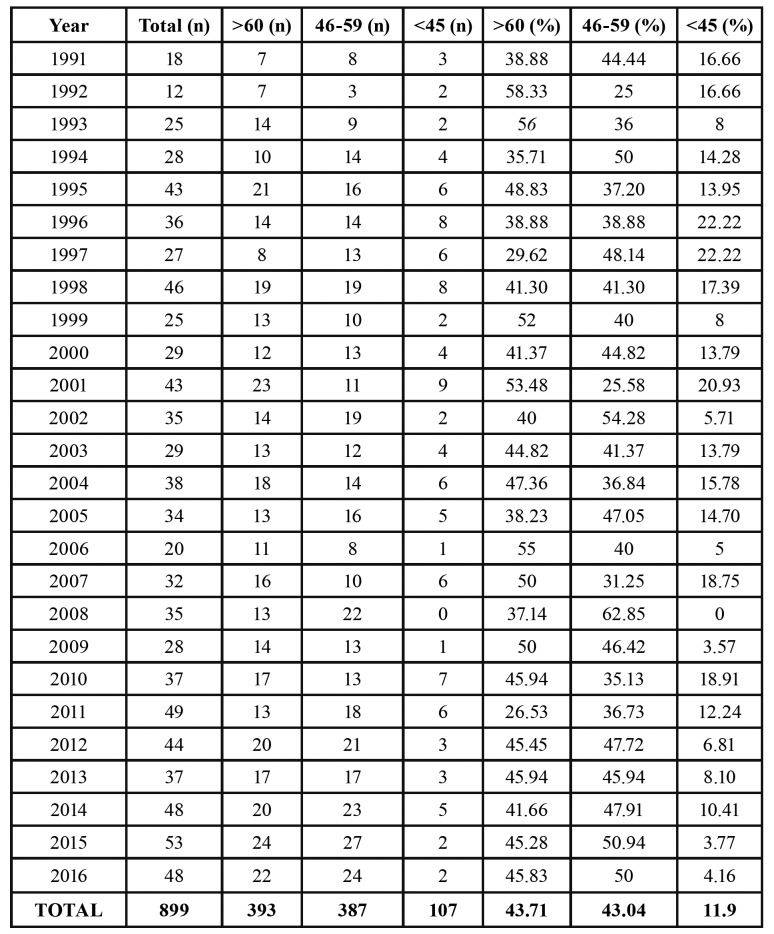
Incidence of HNSCC stratified by year for different age groups.

**Table 2:**
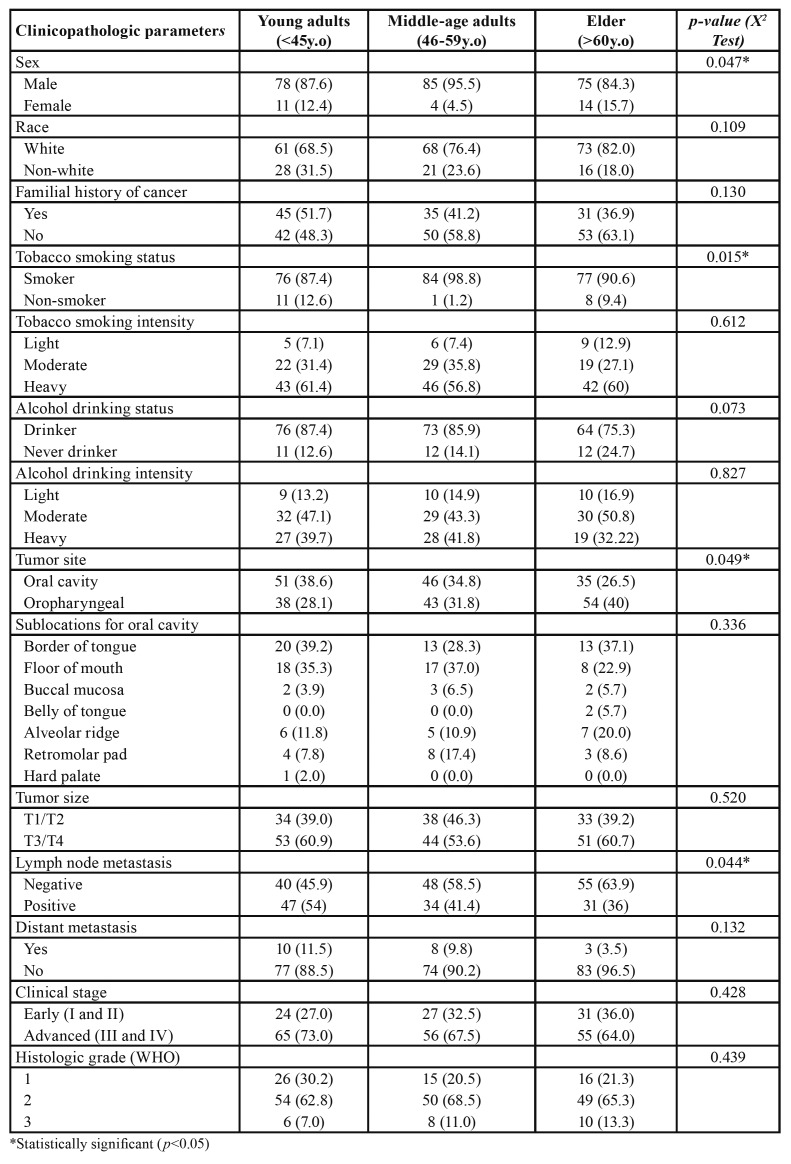
Comparison of clinicopathologic characteristics between young-adults, middle-age adults, and old-aged adults with HNSCC.

- Clinicopathologic characteristics of HNSCC in young patients

The clinicopathologic profile of the HNSCC in the young group was studied separately. A familial history of cancer was significantly associated with the diagnosis of HNSCC in the early stages (*p*= 0.03) (Table 3). Patients with positive status of alcohol consumption were more prone to present with the disease in advanced stages (*p*= 0.03). The same was observed for heavy drinkers (*p*= 0.01) (Table 3).

**Table 3:**
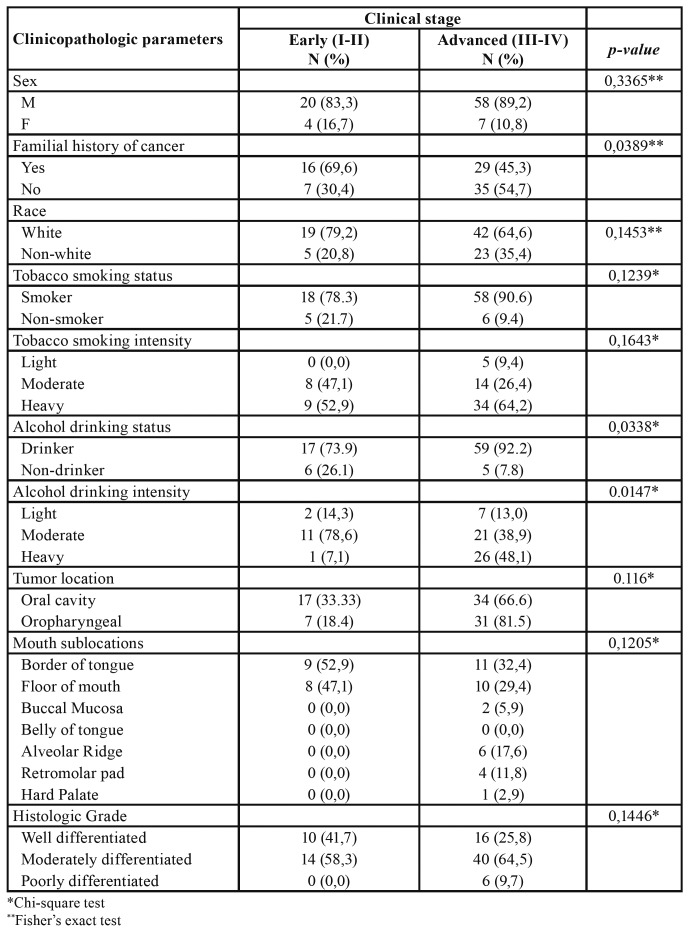
Association of clinicopathologic characteristics to clinical stage of Young patient with HNSCC.

Sex, race, tobacco smoking status and intensity, tumor location and histologic grade were neither associated to initial nor to advanced clinical stages.

We compared the clinicopathologic variables of the young group by tumor location (Table 4). There was no difference between the oral cavity and oropharyngeal tumors regarding sex, familial history of cancer, race, tobacco smoking status, tobacco smoking intensity, alcohol drinking status, tumor size, distant metastasis, and clinical stage. Young patients with oropharyngeal tumors had more lymph node metastasis (*p*<0.01) and poorly differentiated SCC (*p*=0.01). Young patients with oropharyngeal tumors were more likely to be heavy alcohol drinkers (*p*=0.02).

Advanced stages of the HNSCC in young patients were significantly associated with worse survival (*p*= 0.003) (Fig. 1). Survival was not different between young patients with oral cavity and oropharyngeal HNSCC (*p*= 0.7) (Fig. 1). Tobacco smoking, alcohol consumption status, familial history of cancer and histologic grade had no significant influence on the survival of HNSCC young patients.

**Table 4:**
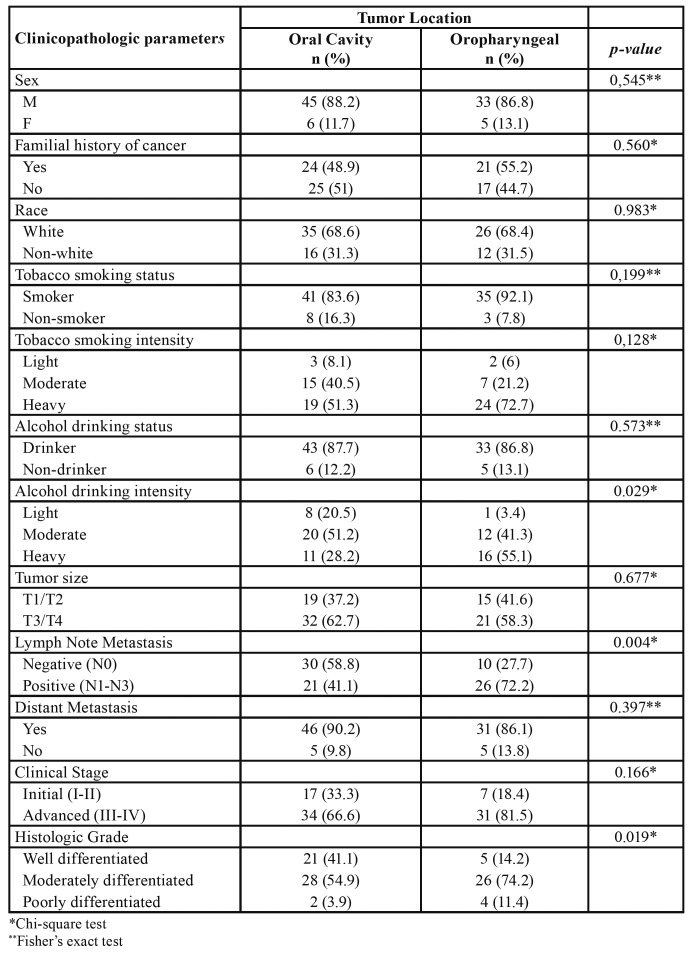
Association of clinicopathologic characteristics to tumor location of Young patient with HNSCC.

## Discussion

In the present 26-year retrospective study accessing data from a single specialized cancer center in the southeast region of Brazil, 899 cases of HNSCC (excluding lips) were found, of which 107 (11.9%) were diagnosed in patients younger than 45-years-old. In general, the oral cavity is the region of the head and neck most commonly affected by SCC (1,2). In the present cohort, young patients were more prone to have oral cavity tumors, while elder patients were mostly affected by oropharyngeal tumors (Table 2). Most studies with young patients have focused the analysis only in SCC of the oral cavity (5). In the current study, there was no significant difference among the age groups regarding sublocations of the oral cavity, which is in accordance with previous investigations (19,20). In contrast, Troeltzsch *et al*. (17) observed that the oral tongue was significantly more affected in the young group than in the middle-aged and elder groups.

The incidence of oral SCC (OSCC) in young people is relatively low and varies depending on the geographic region (5). Estimates for OSCC in people <45-years-old for the North and South Americas are respectively 5.4% and 5.7% (5) of all OSCC cases. The incidence of OSCC found in the present study corroborates with further Brazilian cohorts (11,13) and approximates to cohorts from Africa (17.2%) and Middle East countries (14.5%) (5). Increases in the incidence of OSCC in young people have been observed in several regions, especially in Western countries, along with a decrease in the incidence rates for people of the classic profile for this disease (5). However, an important decrease in the prevalence of young people with OSCC along the last years was observed in a recent Brazilian report (13). The lack of constant decrease and increase in the incidence of HNSCC in young people observed in the present study does not confirm this data. Wider epidemiological multicenter studies are required to clarify the regions in which there is a growth in the incidence of HNSCC in young people.

HNSCC is known to have a notorious predilection for occurring in men (2). Therefore, the female group might be the target for deeper investigations. We observed that the proportion of female patients with HNSCC was significantly higher in the young and elderly groups than in the middle-aged group. In France, Blanchard *et al*. (21) did not find a significant difference between age-groups regarding sex, similar to two previous Brazilian cohorts (19,20). These studies did not segregate the sample in three age groups as in the present study, thus young patients were only compared to patients >45-years-old. However, Santos *et al*. (13), demonstrated that in a population from the northwest region of Brazil, the proportion of female patients in the young group (18.4%) was higher than in the present cohort (12.4%), while Troeltzsch *et al*. (17) did not observe significant differences regarding sex among three age groups in Germany. We believe that the difference in sex distribution among the age-groups observed in the present study is mostly influenced by the higher occurrence of HNSCC in elderly women. The HNSCC in elderly women is also believed to be a distinct disease by some researchers, and is recently being target for deeper investigation, including in our center.

Epidemiological trends for HNSCC in young people might be determined by a distinct etiologic background (5-7). Although most patients in all groups of the current study were smokers, more patients from the young group were never exposed to this habit (Table 2), corroborating with other studies from Brazil (9), The Netherlands (14), and China (22). Toporcov *et al*. (23) did not observe any differences in alcohol and tobacco consumption between young and older patients. However, attribuTable fractions (AF) for smoking and drinking were lower for young patients, suggesting that differences in etiologic factors for HNSCC may indeed exist (23). Curiously, we did not observe a significant association of the status of tobacco smoking with the disease stage for young patients, which was observed for alcohol drinking status and intensity. Santos *et al*. (13) observed a significant association of harmful habits (tobacco and alcohol consumption) with advanced clinical stages of young patients with OSCC, however, these factors were not evaluated separately. Nevertheless, due to the short time of exposure, there remain doubts regarding the role of tobacco and ethanol as risk factors for HNSCC in young people (24).

Although most of the young patients were exposed to the classic risk factors for HNSCC, the risk factors for this disease and its progression in this specific group is not fully compatible with those for older patients, deserving deeper investigations. The only study that investigated in molecular level the expression of enzymes involved in the biotransformation of tobacco and alcohol did not demonstrate great differences between young and older patients (24). However, the pathways involved in the carcinogenesis induced by alcohol and tobacco in young patients still require investigations.

Halboub *et al*. (25) observed that young patients were significantly more prone to have lymphatic metastasis at the time of diagnosis and poorly differentiated OSCC. In the present cohort, although we did not observe differences regarding the histologic grade of tumors, the young patients had more lymph node metastasis than the other groups. Although tumor size and histologic grade of the primary tumor were not different for young patients, the tumors of this group seem to have a higher potential to spread to local lymph nodes. The evidence regarding the biological behavior of HNSCC in young patients is scarce, however, VEGF-C, Podoplanin, and MMP-9 were more expressed in OSCC of young patients in previous studies (26-28). Although these proteins are involved in lymphatic growth and local invasion, neither clinical stage nor lymph node status was different for young patients, and lymph node positivity was not dependent on the overexpression of these proteins (26-28). Further investigations should focus on the tumor lymph node spread mechanisms in young patients.

There is no consensus if the HNSCC in young people has a more aggressive behavior than in older people. Naz *et al*. (29), Blanchard *et al*. (21), and Frare *et al*. (19) neither found significant differences in the clinical parameters of tumors nor differences in the histological grading of tumors between young and older patients, corroborating with our findings. Although in a study by Kaminagakura *et al*. (24) there was no discrepancy between age groups regarding clinical staging, the young patients had more undifferentiated OSCC than the older patients, as also observed by Halboub *et al*. (25). The treatment decision for HNSCC is based mostly on the clinical staging classification. Nevertheless, although many studies show that the clinical stage for young patients is not different from older patients, the molecular profile of tumors in this population might reflect a distinct entity (6) and requires further investigation in order to enable the treatment individualization.

Despite the recent efforts to promote early detection, most HNSCC patients still experiment poor survival (1,2). Frare *et al*. (19) indicated that young patients with HNSCC had a worse prognosis than older patients. On the other hand, Camillon *et al*. (30) observed a significant worsening of survival along with the increasing of age in patients with SCC of the oropharynx. In a study by Van Monsjou *et al*. (14) patients older than 40-years old had worse overall survival than patients younger than 40-years old. In the present cohort, there were no significant differences either on overall survival or on disease-specific survival among the three age groups, in agreement with Zhang *et al*. (22). Worse survival for young patients with HNSCC is believed to be due to differences in the etiologic background (6). However, although in this study we observed that young patients were less exposed to tobacco smoking, this group did not have worse survival, and neither tobacco smoking nor alcohol drinking influenced survival.

Our findings show that young patients with HNSCC were less prone to be smokers than middle-aged and elder patients. The survival profile for the young group was similar to the older groups. Multicenter studies are required to clarify geographic and cultural influences on the occurrence of HNSCC in the young population. In addition, the role of further etiologic factors and tumor behavioral biomarkers must be judiciously studied in the young population with HNSCC, in order to allow better prevention, treatment strategies, and survival improvement.

## References

[1] Bray F, Ferlay J, Soerjomataram I, Siegel RL, Torre LA, Jemal A (2018). Global cancer statistics 2018: GLOBOCAN estimates of incidence and mortality worldwide for 36 cancers in 185 countries. CA Cancer J Clin.

[2] Siegel RL, Miller KD, Jemal A (2017). Cancer Statistics, 2017. CA Cancer J Clin.

[3] van Monsjou HS, Wreesmann VB, van den Brekel MW, Balm AJ (2013). Head and neck squamous cell carcinoma in young patients. Oral Oncol.

[4] Simard EP, Torre LA, Jemal A (2014). International trends in head and neck cancer incidence rates: differences by country, sex and anatomic site. Oral Oncol.

[5] Hussein AA, Helder MN, de Visscher JG, Leemans CR, Braakhuis BJ, de Vet HC (2017). Global incidence of oral and oropharynx cancer in patients younger than 45 years versus older patients: A systematic review. Eur J Cancer.

[6] dos Santos Costa SF, Brennan PA, Gomez RS, Fregnani ER, Santos-Silva AR, Martins MD (2018). Molecular basis of oral squamous cell carcinoma in young patients: Is it any different from older patients?. J Oral Pathol Med.

[7] Liu X, Gao XL, Liang XH, Tang YL (2016). The etiologic spectrum of head and neck squamous cell carcinoma in young patients. Oncotarget.

[8] Martel M, Alemany L, Taberna M, et al (2017). The role of HPV on the risk of second primary neoplasia in patients with oropharyngeal carcinoma. Oral Oncol.

[9] Kaminagakura E, Villa LL, Andreoli MA, et al (2012). High‐risk human papillomavirus in oral squamous cell carcinoma of young patients. Int J Cancer.

[10] Young D, Xiao CC, Murphy B, Moore M, Fakhry C, Day TA (2015). Increase in head and neck cancer in younger patients due to human papillomavirus (HPV). Oral Oncology.

[11] Ribeiro ACP, Silva ARS, Simonato LE, Salzedas LMP, Sundefeld MLMM, Soubhia AMP (2009). Clinical and histopathological analysis of oral squamous cell carcinoma in young people: a descriptive study in Brazilians. Brit J Oral Maxillofac Surg.

[12] Kaminagakura E, Vartanian JG, da Silva SD, dos Santos CR, Kowalski LP (2010). Case‐control study on prognostic factors in oral squamous cell carcinoma in young patients. Head Neck.

[13] Santos HBP, dos Santos TKG, Paz AR, et al (2016). Clinical findings and risk factors to oral squamous cell carcinoma in young patients: A 12-year retrospective analysis. Med Oral Patol Oral Cir Bucal.

[14] van Monsjou HS, Lopez-Yurda MI, Hauptmann M, van den Brekel MW, Balm AJ, Wreesmann VB (2013). Oral and oropharyngeal squamous cell carcinoma in young patients: the Netherlands Cancer Institute experience. Head Neck.

[15] Sivars L, Tani E, Näsman A, Ramqvist T, Munck-Wikland E, Dalianis T (2016). Human papillomavirus as a diagnostic and prognostic tool in cancer of unknown primary in the head and neck region. Anticancer Res.

[16] Hilly O, Shkedy Y, Hod R, Soudry E, Mizrachi A, Hamzany Y (2013). Carcinoma of the oral tongue in patients younger than 30 years: comparison with patients older than 60 years. Oral Oncol.

[17] Troeltzsch M, Knösel T, Eichinger C, et al (2014). Clinicopathologic features of oral squamous cell carcinoma: do they vary in different age groups?. J Oral Maxillofac Surg.

[18] Harris SL, Kimple RJ, Hayes DN, Couch ME, Rosenman JG (2010). Never‐smokers, never‐drinkers: Unique clinical subgroup of young patients with head and neck squamous cell cancers. Head Neck.

[19] Frare JC, Sawazaki-Calone I, Ayroza-Rangel AL, Bueno AG, de Morais CF, Nagai HM (2016). Histopathological grading systems analysis of oral squamous cell carcinomas of young patients. Med Oral Patol Oral Cir Bucal.

[20] Kaminagakura E, Werneck da Cunha I, Soares FA, Nishimoto IN, Kowalski LP (2011). CCND1 amplification and protein overexpression in oral squamous cell carcinoma of young patients. Head Neck.

[21] Blanchard P, Belkhir F, Temam S, El Khoury C, De Felice F, Casiraghi O (2017). Outcomes and prognostic factors for squamous cell carcinoma of the oral tongue in young adults: a single-institution case-matched analysis. Eur Arch Otorhinolaryngol.

[22] Toporcov TN, Znaor A, Zhang ZF, Yu GP, Winn DM, Wei Q (2015). Risk factors for head and neck cancer in young adults: a pooled analysis in the INHANCE consortium. Int J Epidemiol.

[23] Zhang YY, Wang DC, Su JZ, Jia LF, Peng X, Yu GY (2017). Clinicopathological characteristics and outcomes of squamous cell carcinoma of the tongue in different age groups. Head Neck.

[24] Kaminagakura E, Caris A, Coutinho-Camillo C, Soares FA, Takahama-Júnior A, Kowalski LP (2016). Protein expression of CYP1A1, CYP1B1, ALDH1A1, and ALDH2 in young patients with oral squamous cell carcinoma. Int J Oral Maxillofac Surg.

[25] Halboub E, Al-Mohaya M, Abdulhuq M, Al-Mandili A, Al-Anazi Y (2012). Oral squamous cell carcinoma among Yemenis: onset in young age and presentation at advanced stage. J Clin Exper Pent.

[26] de Oliveira Moura JMB, Câmara ACDSM, Nonaka CFW, Pinto LP, de Souza LB (2016). Immunohistochemical comparative analysis of lymphatic vessel density and VEGF-C expression in squamous cell carcinomas of the tongue between young and old patients. Pathol Res Pract.

[27] Sgaramella N, Lindell Jonsson E, Boldrup L, Califano L, Coates PJ, Tartaro G (2016). High expression of podoplanin in squamous cell carcinoma of the tongue occurs predominantly in patients ≤40 years but does not correlate with tumour spread. The J Pathol Clin Res.

[28] Miranda-Galvis M, Santos-Silva AR, Freitas-Jardim J, Paiva-Fonseca F, Lopes MA, de Almeida OP (2018). Different patterns of expression of cell cycle control and local invasion‐related proteins in oral squamous cell carcinoma affecting young patients. J Oral Pathol Med.

[29] Naz S, Salah K, Khurshid A, Hashmi AA, Faridi N (2015). Head and neck squamous cell carcinoma-comparative evaluation of pathological parameters in young and old patients. Asian Pac J Cancer Prev.

[30] Camilon PR, Stokes WA, Nguyen SA, Lentsch EJ (2014). The prognostic significance of age in oropharyngeal squamous cell carcinoma. Oral Oncol.

